# Wohlfahrtiimonas chitiniclastica Bacteremia: A Rare Case of a Male With Maggot-Infested Lower Extremity Wounds

**DOI:** 10.7759/cureus.66711

**Published:** 2024-08-12

**Authors:** Sina Hedayatpour, Ryan Parkinson, Deepak Bommisetty

**Affiliations:** 1 Internal Medicine, Methodist Health System, Dallas, USA

**Keywords:** osteomyelitis, maggot, wound, bacteremia, wohlfahrtiimonas chitiniclastica

## Abstract

*Wohlfahrtiimonas chitiniclastica* (*W. chitiniclastica*) is an emerging gram-negative bacillus rarely found in patients presenting with fly myiasis or parasitic larvae infection. Here, we present the case of a 58-year-old male who presented with *W. chitiniclastica* bacteremia from lower extremity wounds complicated by fly larvae infestation. Blood cultures were analyzed with matrix-assisted laser desorption ionization-time of flight mass spectrometry, which identified *W. chitiniclastica*. The patient was treated with empiric antibiotic therapy with piperacillin-tazobactam and de-escalated to ceftriaxone. We discuss the potential impact of environmental interactions with zoonotic vectors and the concern for the increasing incidence of this new emerging zoonotic infection. This appears to be the first reported case of *W. chitiniclastica* bacteremia in the southern United States and demonstrates a growing list of climates and locations in which this organism can be present. Further evaluation of potential vectors for *W. chitiniclastica* continues to be a priority for how cases are distributed and can present in patients.

## Introduction

*Wohlfahrtiimonas chitiniclastica* (*W. magnifica*)is an aerobic, nonmotile, gram-negative bacillus that was first isolated from third-stage fly larvae of the obligate parasitic fly *Wohlfahrtia magnifica* in 2008 in Hungary [1]. *W. magnifica* is documented as a livestock pest in Eastern Europe, the Mediterranean, and Middle Asia. *W. magnifica* and other obligate parasitic flies deposit eggs and larvae in the wounds of humans and other vertebrates, a process called myiasis. In 2009, the first published case of *W. chitiniclastica* documented the then-unknown gram-negative bacillus in a larvae-infested homeless woman's blood cultures in France [2]. The first case of fulminant sepsis due to *W. chitiniclastica* was reported in 2011 in a homeless Argentinian male who developed septic shock and multiorgan failure resulting in a fatal outcome [3]. Although no larvae were reported, the patient had multiple erythematous plaques with honey-colored crusts in the inguinal area that were speculated to have been entry wounds for larvae. These initial cases were the foundation for further case reports tracking this pathogen and further detailed studies into the organism.

*W. chitiniclastica* infection typically presents in patients with necrotic and larva-infested wounds, poor hygiene conditions, polymicrobial infection, and/or osteomyelitis. Treatment is often dependent on antibiotic susceptibilities and source control. Based on resistance profiles, *W. chitiniclastica* is sensitive to beta-lactam antibiotics such as penicillins, cephalosporins, and carbapenems [4]. In a literature review by Kopt et al., 43 case reports of *W. chitiniclastica* in humans were reported as of March 2023 [4]. Of those cases, 18 had documented bacteremia. In this case report, we present a homeless male with a maggot-infested wound that resulted in *W. chitiniclastica* bacteremia.

## Case presentation

**Figure 1:**
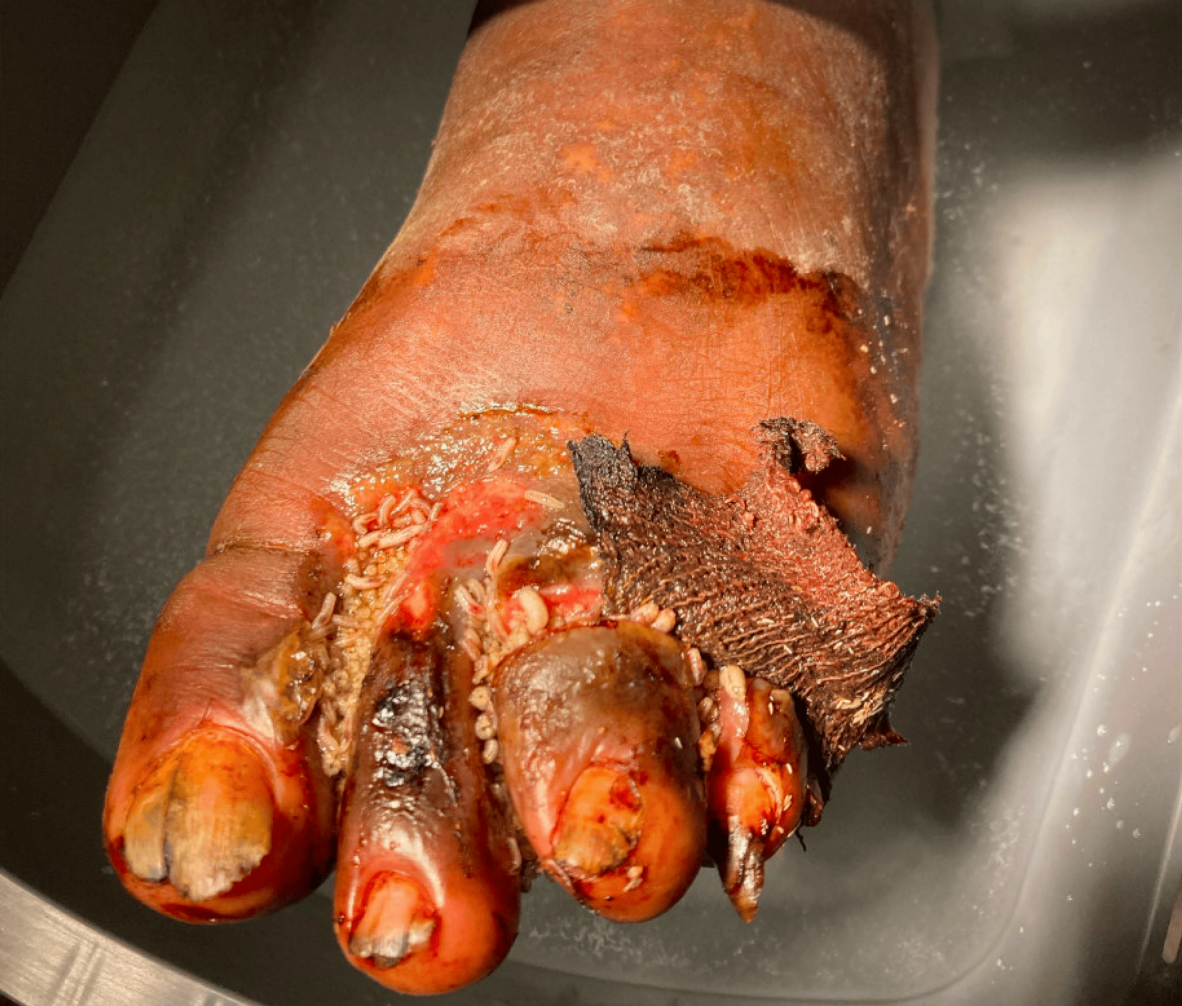
Clinical appearance of a maggot-infested wound of the patient’s left lower extremity

A 58-year-old homeless male with a past medical history of bipolar disorder presented with a left foot wound with associated pain for a few weeks prior to presentation. The patient had noted maggots on the left foot wound for a few days. Initial vital signs included a temperature of 36.5°C, a heart rate of 95 bpm, and a blood pressure of 144/88 mmHg. On physical exam, the patient had a maggot-infested wound of the lower left leg in both the dorsal and plantar surface, mostly involving the interdigital space as well as across the third digit (Figure 1). His initial white blood cell count was within normal limits at 7.0 x 10^3^/µL, but the inflammatory markers C-reactive protein and erythrocyte sedimentation rate were elevated at 21 mg/L and 38 mm/hr, respectively. An initial x-ray of the left foot noted lytic changes within the proximal phalanx of the third digit, suggesting osteomyelitis. The wound care staff debrided the patient's wounds and discarded the maggots. Blood cultures were notable for multiple pathogens, including methicillin-sensitive *Staphylococcus aureus*, *Providencia rettgeri*, and an aerobic gram-negative nonfermenter species that was later determined to be *W. chitiniclastica* through matrix-assisted laser desorption ionization-time of flight (MALDI-TOF) mass spectrometry. Infectious disease and podiatry services were consulted. A magnetic resonance imaging scan was indicative of a third-digit osteomyelitis. The patient was treated with empiric piperacillin-tazobactam and transitioned to ceftriaxone after susceptibilities were available. He underwent a bone biopsy per recommendations from podiatry, which was negative for osteomyelitis, and amputation was deferred. The patient remained in the hospital to complete a six-week course of IV ceftriaxone inpatient, given his unfunded status and homelessness.

## Discussion

Based on a thorough review of current publications, this is the first reported case of *W. chitiniclastica* bacteremia in Texas, USA. The first case of *W. chitiniclastica* bacteremia in the United States was recorded in Hawaii in 2016, followed by case reports of bacteremia in Ohio and mainly northern states [4]. Furthermore, this case highlights several important points regarding this emerging zoonotic pathogen. Initial laboratory studies identified *W. chitiniclastica* as an aerobic, nonmotile, nonlactose fermenting gram-negative rod that obligate parasitic flies transmit. The first described vector was *Wohlfahrtia magnifica*. Other known vectors include *Lucilia sericata*, *Musca domestica*, and *Chrysomya megacephala* [5]. Expert opinion suggests that *W. chitiniclastica* maintains a symbiotic relationship with parasitic fly larvae by helping them digest chitin with chitinase, an enzyme that helps break down the polysaccharide found in fungal cell walls and exoskeletons of certain animals [5]. This enzymatic activity plays a role in the larvae's metamorphosis, helping the insect develop into its adult form. Another unique characteristic of *W. chitiniclastica* is its association with myiasis; the genus *Ignatzschineria* is the only other well-known bacterial species associated with myiasis [6]. Interestingly, *W. chitiniclastica* has also been discovered in other habitats, such as arsenic-contaminated soil, in the pancreas of a zebra, in frozen chicken meat, in aquatic plants, and in the human gut microbiome of deceased individuals [7-11]. Whether or not these findings reflect other routes of transmission remains unknown.

Four cases of *W. chitiniclastica* infection were reported to have a fatal outcome, all of which had confirmed blood culture growth of *W. chitiniclastica *[4]. The most common risk factors were chronic or necrotic wounds and ulcers, cardiovascular disease, and poor hygiene conditions. Notably, nearly two-thirds of the cases did not report larvae or infested wounds. This finding indicates that wound larvae infestation may have occurred prior to presentation or was overlooked on initial physical exam. In one case report, *W. chitiniclastica* bacteremia was associated with maggot debridement therapy, highlighting a potential risk associated with this treatment [12]. *W. chitiniclastica* bacteremia is typically associated with polymicrobial infection, which can further complicate the diagnosis and identification of this disease. Further studies need to be conducted to clarify if this organism is a disease-causing pathogen or a part of polymicrobial infection.

Identification of *W. chitiniclastica* is securely identified through MALDI-TOF or 16s rRNA gene sequencing [13]. Given the increasing use of these technologies, it is expected that identification of this bacteria will continue to increase. *W. chitiniclastica* may also be affected by changes in climate. Further epidemiological studies are needed to examine potential vectors and incidence rates. Treatment of *W. chitiniclastica* is often based on the organism's susceptibility. As a gram-negative rod that is not often exposed to antibiotics naturally, empiric coverage for soft tissue infection and osteomyelitis is presumed to be adequate for *W. chitiniclastica*. In the majority of case reports, *W. chitiniclastica* species were sensitive to and treated with beta-lactam antibiotics such as penicillins, cephalosporins, and carbapenems. Other options include fluoroquinolones, with levofloxacin being the more favorable agent of this class [4].

## Conclusions

This case highlights the importance of clinicians recognizing *W. chitiniclastica* as a pathogen directly linked to myiasis. As such, identifying this pathogen should alert providers of the presence of larvae-infested wounds if they are not apparent on initial presentation. This pathogen has been discovered in various climates and is likely as ubiquitous as the larvae vectors it inhabits. Clinicians should be aware of common underlying conditions linked to *W. chitiniclastica* infection, such as chronic wounds, poor hygiene, and low socioeconomic status. With the increase in the world population and interaction with animals, more cases of *W. chitiniclastica* infection will likely be reported.
